# Prenatal Genetic Counseling in a Chinese Pregnant Woman With Rare Thalassemia: A Case Report

**DOI:** 10.3389/fgene.2021.670168

**Published:** 2021-05-28

**Authors:** Liangying Zhong, Ye Wang, Wenbin Lin, Zhenrong Yao, Jiang Zhang, Hongxu Xu, Pinning Feng, Lijuan Xu

**Affiliations:** ^1^Department of Laboratory Medicine, The First Affiliated Hospital, Sun Yat-sen University, Guangzhou, China; ^2^Fetal Medicine Center, Department of Obstetrics and Gynecology, The First Affiliated Hospital, Sun Yat-sen University, Guangzhou, China; ^3^Department of Endocrinology, The First Affiliated Hospital, Sun Yat-sen University, Guangzhou, China

**Keywords:** thalassemia, HbJ-Bangkok, IVS-II-806(G>C), deep intronic variant, case report

## Abstract

**Background:** Prenatal genetic counseling can be difficult, especially when it is related to fetuses with a rare thalassemia. An intronic variant located far from obvious regulatory sequences in the *HBB* gene could be very difficult to evaluate as it may affect the mRNA processing or cause β-thalassemia (β-thal). In the present study, a Chinese pregnant woman with HbJ-Bangkok and a very rare change in the second intron of the *HBB* gene [IVS-II-806(G>C), NM_000518.4, *HBB*: c.316-45G>C] in combination with α+-thalassemia was reported, which can assist in prenatal genetic counseling.

**Case Report:** A 26-year-old pregnant woman presented at the obstetric clinic for a routine pregnancy check at 12 weeks of gestation. Red blood counts and high-performance liquid chromatography (HPLC) were consistent with clinical manifestations of anemia. Multiplex gap-polymerase chain (gap-PCR) displayed rightward deletion (–α^3.7^/αα). Direct DNA sequencing of the δ-globin gene showed no mutation. Sanger sequencing of the β-globin gene showed a previously undescribed condition of double heterozygosity for HbJ-Bangkok and a very rare change in the second intron of the *HBB* gene [IVS-II-806(G>C), NM_000518.4, *HBB*: c.316-45G>C] that has not been previously reported in the HbVar database. Thus, a rare combination of α+-thal and a compound heterozygosity of HbJ-Bangkok and [IVS-II-806(G>C)] with α+-thal (–α^3.7^/αα) was finally diagnosed. Prenatal genetic counseling was made based on the genotype and phenotype analyses.

**Conclusion:** This study enlarges the mutation spectrum of β-globin gene and emphasizes DNA analysis in resolving unusual patterns in Hb analysis and the importance of sharing the observed rare undefined mutations and the possible interactions with known molecular defects, which can assist in prenatal genetic counseling.

## Introduction

Beta-thalassemia characterized by insufficient or missing synthesis of the β-globin chain of hemoglobin is an autosomal recessive disorder (Origa, 2017). To date, more than 1,000 hemoglobin (Hb) variants have been reported (Giardine et al., 2014). Alpha-thalassemia, which is characterized by the decrease or complete suppression of α-globin chains, is an autosomal recessive genetic defect that is frequent in Southeast Asian countries (Sengchanh et al., 2005; Celik et al., 2013). It can be divided into α+-thalassemia and α0-thalassemia. The most common form of α+-thalassemia in populations is the 3.7-kb deletion (–α^3.7^) (Charoenwijitkul et al., 2019).

To the best of our knowledge, there are few reports of coinheritance of α+-thalassemia with compound heterozygosity of β-globin gene mutation and β-globin variant. We herein report a β-thalassemia patient with a normal level of HbA2 who had a point mutation in the intron 2 region of the β-globin gene [IVS-II-806(G>C), NM_000518.4, *HBB*: c.316-45G>C] combined with HbJ-Bangkok associated with the α+-thal (–α^3.7^/αα) mutation. Hematological indexes of this patient are presented and compared with those of simple heterozygotes for HbJ-Bangkok, to help better understand the genetic and clinical characteristics of this variant as well as to provide information for genetic counseling.

## Case Report

We present a 26-year-old pregnant woman from Chengdu City, Sichuan Province, China, who was referred to the obstetric clinic for a routine pregnancy check in the first trimester at 12 weeks of gestation. Hb electrophoresis and conventional peripheral blood counts were determined during our prenatal screening program. The patient's blood sample and those of her parents and husband were collected using EDTA as an anticoagulant. Clinical examinations showed that she had mild anemia (Hb level of 118 g/L), as well as hematological features of hypochromic microcytosis with a mean cell hemoglobin (MCH) of 26.7 pg and a mean cell volume (MCV) of 78.9 fl. Iron deficiency was excluded. Her husband had normal Hb and normal MCV and MCH.

**Figure 1:**
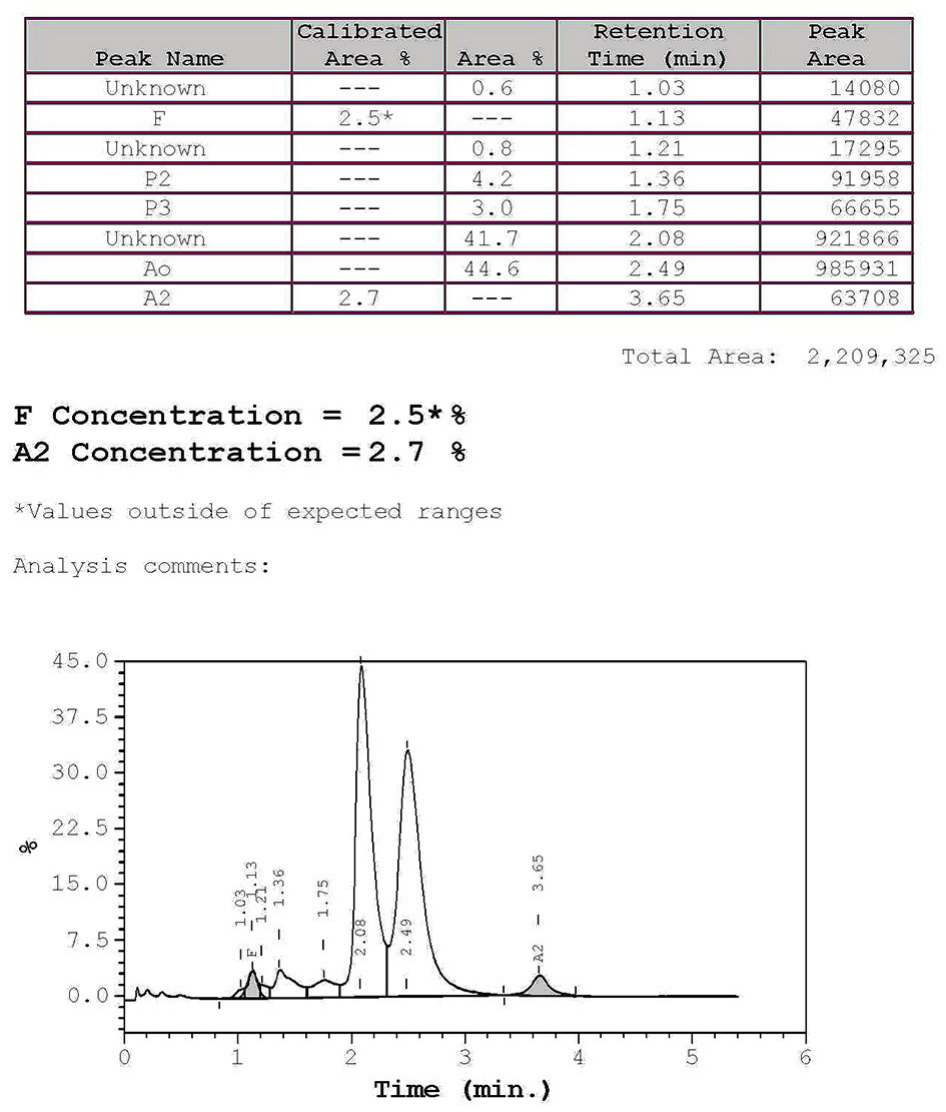
High-performance liquid chromatography (HPLC): hemoglobin analysis of the patient. The percentages of HbA2 and HbF were 2.7 and 2.5%, respectively, and the abnormal peak was 41.7%, and HbA0 was 44.6% as determined by hemoglobin electrophoresis.

**Figure 2:**
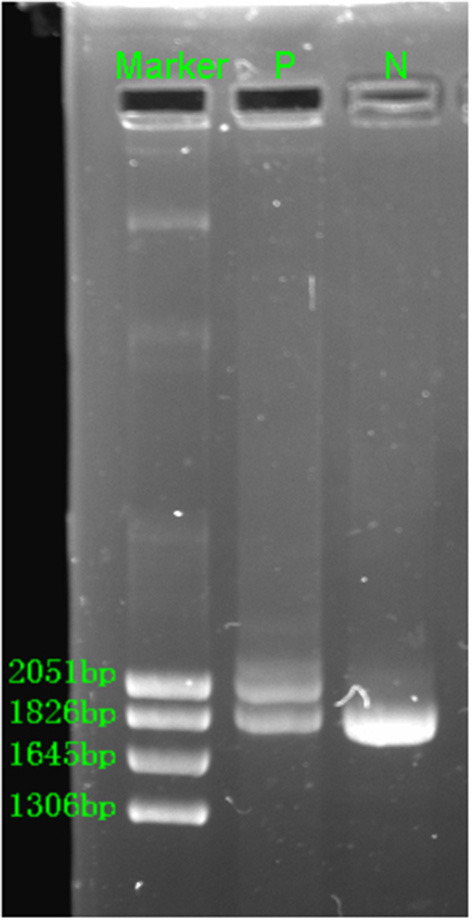
Gap-PCR revealed the rightward deletion (–α^3.7^/αα) for the patient. N, normal; P, patient.

**Figure 3:**
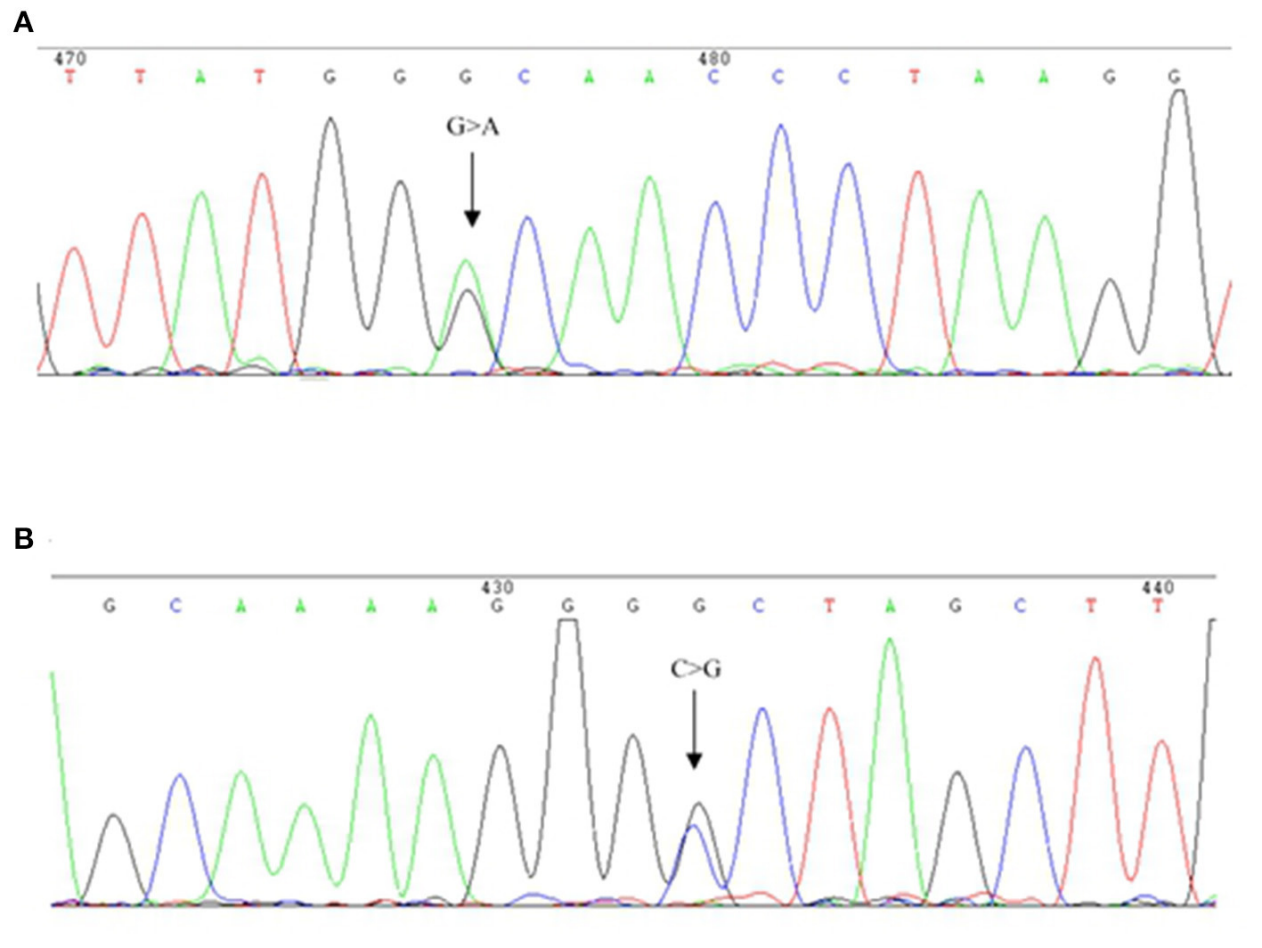
DNA sequences of patient β-globin genes. **(A)** HbJ-Bangkok (GGC → GAC) substitution at the β-globin gene was observed (indicated by an arrow). **(B)** Part of the reverse DNA sequencing of the β-globin gene showing the heterozygous mutation *HBB*: c.316-45G>C (indicated by an arrow).

The hematological indexes are summarized in Table 1. The erythrocytes of the patient exhibited hypochromia and mild microcytosis. The Hb electrophoresis results showed that the levels of HbA2 were 2.7%, HbF 2.5%, and HbA0 44.6%, and an abnormal unknown peak of HbX (41.7%) (Figure 1) appeared at a retention time (RT) of 2.08 min. Therefore, the low MCV/MCH values, the normal HbA2 levels, and the clinical phenotype and red cell abnormalities were inconsistent with the Hb electrophoresis results. Gap-PCR revealed a heterozygote of the –α^3.7^ deletion (–α^3.7^/αα) (Figure 2) of the patient, while the RDB assay for the α2 gene mutations indicated no mutations. The β-thalassemia point mutation RDB assay for the 17 genotypes (Cai et al., 1994) showed no mutation. The δ-globin gene which may reduce the increased HbA2 levels of β-thalassemia to normal was sequenced and displayed no mutation. Sanger sequencing of the β-globin gene confirmed compound heterozygosity for HbJ-Bangkok (Figure 3A) and a mutation in the intron 2 region [IVS-II-806(G>C)] (Figure 3B). Thus, the patient had a combination of α+-thal (–α^3.7^/αα) and a compound heterozygote of HbJ-Bangkok and a mutation in the β-globin gene [IVS-II-806(G>C)]. Clinical details and clinical images were obtained from the participant.

**Table 1:** Hematological features, Hb electrophoresis, and genotype analysis of the patient.

**Test**	**Result**	**Reference interval**
Sex/age (years)	F/26	–
RBC (×10^12^/L)	4.6	4.0–5.5
Hb (g/L)	118	120–160
MCV (fl)	78.9	82–95
MCH (pg)	26.7	27–31
MCHC (g/L)	338	320–360
RDW (%)	13	12–15
WBC (×10^12^/L)	10.29	4.0–10.0
NEUT (×10^9^/L)	0.588	0.460–0.750
LYMPH (×10^9^/L)	0.309	0.190–0.470
MONO (×10^9^/L)	0.058	0.030–0.080
EOS (×10^9^/L)	0.36	0.005–0.050
BASO (×10^9^/L)	0.001	0.000–0.010
PLT (×10^12^/L)	292	100–300
Ferritin (μg/L)	18	16.4–323
EPO (IU/L)	18.77	3.7–31.5
HbA2 (%)	2.7	2.5–3.5
HbA0 (%)	44.6	≥80
HbX (%)	41.7	–
HbF (%)	2.5	0.0–2.3
RDW (%)	14	12–15
α genotype	–α^3.7^/αα	–
β genotype	β^HbJ−Bangkok^/β^IVS−II−806^	–

*Hb, hemoglobin; RBC, red blood cell; RDW, red cell distribution width; F, female; MCV, mean cell volume; MCH, mean cell hemoglobin; MCHC, mean cell hemoglobin concentration*.

Early pregnancy was uneventful except for subclinical hyperthyroidism. In a review of patients from previously reported publications to date and from our study here, the molecular and hematological characteristics of HbJ-Bangkok heterozygosity or HbJ-Bangkok complicated with other types of thalassemia are summarized in Table 2.

**Table 2:** Molecular and hematological characteristics of HbJ-Bangkok (*HBB*: c.170G>A) heterozygosity or complications with other types of thalassemia.

**Hb variant**	**Race**	**Hb**	**MCV**	**MCH**	**HbA2**	**HbX**	**α genotype**	**β genotype**	**References**
J-Bangkok	Thai	81	63	17.3	0.2	35.7	–/α^CS^α	None	Fucharoen et al., 2001
J-Bangkok	Tunisian	136	91.5	29.9	2.5	51.8	None	None	Xu et al., 2004
J-Bangkok	Chinese	142	75.9	25.5	2.6	44.8	–α^Q−*Thailand*^/–α^4.2^	None	Jiang et al., 2016
J-Bangkok	Thai	123	79.7	26.9	None	67.7	None	HbE	Fucharoen et al., 2005
J-Bangkok	Chinese	114	61	20.4	4	23.6	Hb G-Honolulu	β^CD41−42^β^N^/ββ	Chang et al., 2002
J-Bangkok	Chinese	110	66.7	19.4	4.7	90.4	None	β^IVS−II−654^β^N^/ββ	Zhao et al., 2013
J-Bangkok	Chinese	118	78.9	26.7	2.7	41.7	–α^3.7^/αα	β^IVS−II−806^β^N^/ββ	This study

## Discussion and Conclusion

To further investigate the underlying mutation in the patient, the PCR product of the β-globin gene was analyzed by direct DNA sequencing. A previously undescribed condition of double heterozygosity for HbJ-Bangkok and a deep intronic variant in the second intron of the β-globin gene [IVS-II-806(G>C), NM_000518.4, *HBB*: c.316-45G>C] change at 806, a G>C mutation, and a potential 5′ splice site within the IVS-II was found. This deep intronic variant had not been previously reported in the HbVar database (http://globin.bx.psu.edu) but reported (rs140033163) with clinical significance “benign” in dbSNP Short Genetic Variation (https://www.ncbi.nlm.nih.gov/projects/SNP/). Chen et al. have already reported two cases of β^IVS−II−806^ alone in Nanping area of Fujian. However, there were no clinical data such as hematological features or Hb electrophoresis of the two patients. There are geographical and ethnic differences in thalassemia (Yu et al., 2015; Huang et al., 2019). The father of the proband is a heterozygous of HbJ-Bangkok, while the mother has a nt substitution of the β-globin gene IVS-II-806(G>C); therefore, HbJ-Bangkok was in trans with IVS-II-806(G>C).

HbJ-Bangkok (also called HbJ-Manado, HbJ-Korat, and HbJ-Meinung) was first described in a Chinese-Canadian newborn and is occasionally found in Black Americans and Japanese, Taiwanese, and Thai individuals (Clegg et al., 1966; Pootrakul et al., 1970; Honig et al., 1982; Iuchi et al., 1982; Yang et al., 1984). HbJ-Bangkok is caused by heterozygosis for the GGC → GAC substitution at codon 56 of the β-globin chain (Pootrakul et al., 1970). Most patients with HbJ-Bangkok were identified during epidemiological investigations and screening of hemoglobin electrophoresis populations (Xiong et al., 2010; Fucharoen et al., 2011; Lin et al., 2012; Huang et al., 2019). HbJ-Bangkok is clinically asymptomatic and hematologically normal (Clegg et al., 1966). Previous reports in Thailand reported cases with HbJ-Bangkok with HbH disease (Fucharoen et al., 2001) or HbE (Fucharoen et al., 2005), and the clinical manifestation of the patients depended mainly on the type of underlying thalassemia genes. A patient with triple concurrent heterozygosis with α-chain abnormal hemoglobin and β-thal and HbJ-Bangkok was found in Taiwan, China (Chang et al., 2002), and a genotype–phenotype correlation showed that the clinical features for β-thalassemia minor with stable Hb variant were consistent with pure β-thalassemia minor. The clinical manifestations in our patient are consistent with such cases that can be explained by the type of underlying abnormal thalassemia genes.

In our report, the patient showed mild microcytic anemia according to her clinical symptoms: normal HbA2 value (2.7%) and low HbA0 value (44.6%) but an abnormal peak HbX (41.7%) on HPLC electropherogram, which is consistent with a previous study (Jiang et al., 2016). Most patients with α-thalassemia have mild anemia, and the condition may not be detected except when a routine blood test is performed (Harteveld and Higgs, 2010). Alpha-thalassemia patients present variable degrees of anemia, decreased mean corpuscular hemoglobin (MCH), reduced mean corpuscular volume (MCV), and/or possibly a normal or slightly reduced level of HbA2 (Harteveld and Higgs, 2010). Sometimes, carriers of α+-thal have normal hematology results, especially those with –α^3.7^/αα and non-deletional mutations in the α1 gene. Such patients may be borderline hypochromic without anemia or normocytic, which can only be found occasionally during routine examination for hemoglobinopathies (Galanello and Origa, 2010), which is consistent with our study. Beta-thalassemia usually has a high HbA2 level (Mosca et al., 2009) only when β-globin silent mutations, for example, very slight microcytosis, are associated with consistent residual output of Hb beta chains and with normal or borderline HbA2 and normal RBC indexes (Galanello and Origa, 2010). Beta-thalassemia coinheritance of iron deficiency or δ-thal may reduce the increased HbA2 levels typical of β-thalassemia carriers to normal (Giambona et al., 2006; Galanello and Origa, 2010; Chaweephisal et al., 2019). Hemoglobinopathies complicated with other types of thalassemia may present clinical manifestations of thalassemia to various degrees (Xu et al., 2004; Singer, 2009; Xiong et al., 2010; Lin et al., 2012; Zhao et al., 2013).

A deep intronic variant is located far from obvious regulatory sequences and it may be more difficult to evaluate its functional significance (Grimholt et al., 2018). In this study, the nucleotide substitution found in the proband is located in the second intron. In this region, a lot of mutations associated with mild or severe β-thalassemia have been described, such as IVS-II-654 (*HBB*: c.316-197C>T) (Vinciguerra et al., 2014), IVS-II-726(A>G) (*HBB*: c.316-125A>G), and IVS-II-809(–C) (*HBB*: c.316-42delC) (Vinciguerra et al., 2017). In ClinVar (ID: 495995), this mutation was defined as benign/likely benign. However, no experimental evidence demonstrating its impact on protein function has been reported. In this study, we presented the hematological features of complex carriers with this variant. Our data showed that *HBB*: c.316-45G>C in the heterozygous state (the mother of the proband) is clinically silent, with normal MCV (83 fl) and MCH (27.2 pg), and HbA2 (2.6%) values were within the normal range. The proband who confirmed to be a compound heterozygosis of HbJ-Bangkok and *HBB*: c.316-45G>C with α+-thal (-α^3.7^/αα) presented mild microcytic anemia, more likely a performance of –α^3.7^ heterozygote. So, the proband was suggested to continue pregnancy without prenatal puncture. However, we had no cases of homozygosity for the *HBB*: c.316-45G>C mutation. A previous report described two deep intronic variants both firstly reported to cause β-thal and later as benign sequence variants (Grimholt et al., 2018). So, more cases are needed from different races and populations, and especially *in vitro* transient expression studies of the mutant gene are warranted to determine whether it is a silent β-thal allele or a polymorphism.

**Figure 4:**
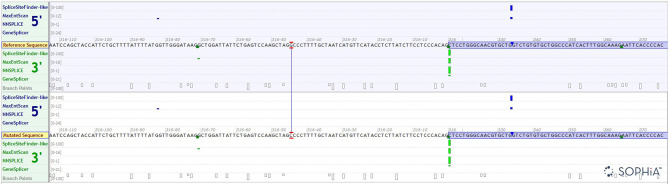
Prediction of the potential effect of *HBB*: c.316-45G>C on splicing by Alamut.

**Figure 5:**
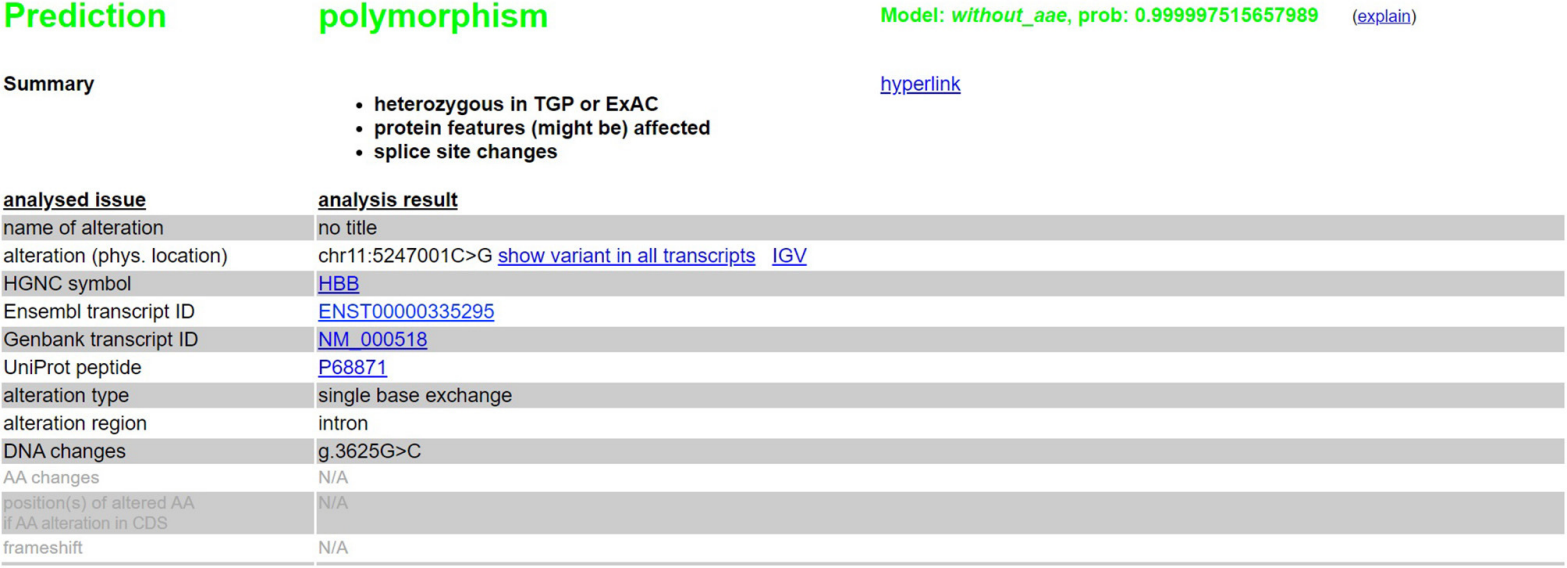
Prediction of the potential effect of *HBB*: c.316-45G>C on splicing by MutationTaster.

**Figure 6:**
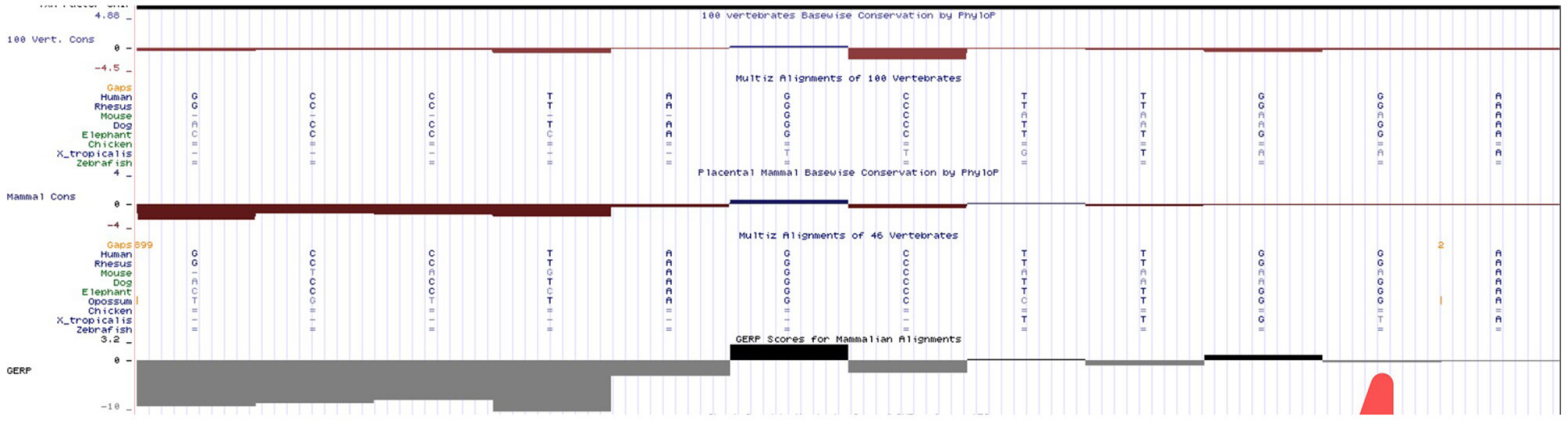
Evolutionary conservation of *HBB*: c.316-45G>C in the UCSC.

We have done an *in silico* analysis to test our assumption that *HBB*: c.316-45G>C is more like a polymorphism than a mutation responsible for a silent β-thalassemia. Prediction of the potential effect of *HBB*: c.316-45G>C on splicing by Alamut (Figure 4) is supplemented, which shows no effect on the splicing site. Moreover, it is predicted by MutationTaster to be polymorphism (Figure 5). Evolutionary conservation in the Genome Browser database of the University of California Santa Cruz (UCSC) (Figure 6) is shown.

The son of the proband is 2 years old now. He was delivered vaginally with a birth weight of 3,150 g. There was no history of jaundice at birth and no obvious retardation of growth and development. The boy was comfirmed to be a heterozygote of *HBB*: 316-45G>C sequence variant alone. He has a hematological feature with Hb level of 121 g/l, MCV of 27.3 fl, MCH of 82.4 pg, and HbA2 of 2.7%. Thus, we consider *HBB*: c.316-45G>C as a benign sequence variant without thalassemic effect.

In conclusion, we present the first hematological parameters in a Chinese patient with compound heterozygosity of HbJ-Bangkok and *HBB*: c.316-45G>C in the intron 2 region of the β-globin gene with α+-thal (–α^3.7^/αα), which behaved as a mild α+-thal trait. This rare mutation is more like a polymorphism than a mutation responsible for a silent β-thalassemia. The clinical and laboratory data of this rare case could be helpful for the evaluation of new cases and for genetic counseling, especially in a region such as Southeast Asia, where the prevalence of thalassemia is very high.

## Data Availability Statement

The raw data supporting the coclusions of this article will be made available by the authors, without undue reservation.

## Ethics Statement

The studies involving human participants were reviewed and approved by the Ethics Committee of the First Affiliated Hospital of Sun Yat-sen University. The patients/participants provided their written informed consent to participate in this study. Written informed consent was obtained from the individual(s) for the publication of any potentially identifiable images or data included in this article.

## Author Contributions

LZ and YW conceived and directed the first draft of the manuscript. ZY and HX collected the clinical data and care for the patient. PF reviewed the literature. WL and JZ reviewed the patients' information and composition of the manuscript. LX guided and designed the research study. All authors have read and approved the final manuscript.

## Conflict of Interest

The authors declare that the research was conducted in the absence of any commercial or financial relationships that could be construed as a potential conflict of interest.

## Supplementary Material

Supplementary Material Methods of hematological analysis and DNA analysis.
